# Supplementary material for: Nonnegative spatial factorization applied to spatial genomics
Source: Nat Methods. 2022 Dec 31;20(2):229–38. doi: 10.1038/s41592-022-01687-w (PMC9911348; doi:10.1038/s41592-022-01687-w)
Supplement: Supplementary file 1 — Supplementary Introduction, Methods, Discussion, Figs. 1–10 and Tables 1–9. [file 41592_2022_1687_MOESM1_ESM.pdf]

nature methods

Article

https://doi.org/10.1038/s41592-022-01687-w

Nonnegative spatial factorization applied to spatial genomics

In the format provided by the authors and unedited

Nonnegative spatial factorization applied to spatial genomics

F. William Townes (email: ftownes@andrew.cmu.edu)[1,2] and Barbara E. Engelhardt (email: barbara.engelhardt@gladstone.ucsf.edu)[1,3,4]

[1]Department of Computer Science, Princeton University, 35 Olden St., Princeton, NJ 08540, USA
[2]Present address: Department of Statistics and Data Science, Carnegie Mellon University, 5000 Forbes Ave., Pittsburgh, PA 15213, USA
[3]Present address: Data Science and Biotechnology Institute, Gladstone Institutes, 1650 Owens St., San Francisco, CA 94158, USA
[4]Present address: Department of Biomedical Data Science, Stanford University, 1265 Welch Road MC5464, MSOB West Wing, Third Floor, Stanford, CA 94305, USA

Contents

1 Supplemental Introduction **2**
- 1.1 Spatial transcriptomics and single-cell RNA-seq 2
- 1.2 Gaussian process models 2
- 1.3 Nonnegativity 3

2 Supplemental Notes **3**
- 2.1 Nonnegative spatial factorization inference 3
  - 2.1.1 Evidence lower bound objective (ELBO) function 3
  - 2.1.2 Parameter estimation 6
  - 2.1.3 Real-valued spatial factorization inference 6
- 2.2 Nonspatial count factorization inference 6
- 2.3 Nonnegative spatial factorization hybrid (NSFH) model 6

3 Supplemental Discussion **7**

4 Supplemental Figures **8**

5 Supplemental Tables **17**

1 Supplemental Introduction

Spatially-resolved transcriptomics (ST) has revolutionized the study of intact biological tissues [1, 2, 3]. In contrast to single-cell RNA sequencing (scRNA-seq), which dissociates cells before sequencing each one, ST quantifies gene expression while preserving the spatial context of the cells within the tissue sample. Since the state and function of each cell is highly dependent upon interactions with its neighbors [4], measuring spatially-resolved transcription represents a crucial advance in our ability to understand cellular state and interactions.

1.1 Spatial transcriptomics and single-cell RNA-seq

Like scRNA-seq, ST data generally consist of discrete counts of transcript fragments from tens to thousands of genes, many of which have zero counts. In both techniques there is typically no ground truth assignment of cell types. There are two basic strategies to measure spatial gene expression: microscopy and bead capture. Microscopy approaches have excellent spatial resolution, even at the sub-cellular level, but require specialized equipment and may not capture large numbers of cells or genes easily. Examples of microscopy protocols include seqFISH+ [5] and MERFISH [6]. Protocols based on bead capture and sequencing tend to have coarser spatial resolution but cover larger spatial areas or larger numbers of genes. They are popular because they use equipment and experimental procedures that are similar to single-cell RNA-seq (scRNA-seq). Examples of bead protocols include high definition spatial transcriptomics (HDST) [7], Slide-seqV2 [8], and the Visium platform from 10x genomics.

Dimension reduction (DR) is a vital tool for unsupervised learning, and there has been a proliferation of DR methods for both scRNA-seq [9, 10, 11] and ST [12, 13]. DR based on a Gaussian error assumption, such as principal components analysis (PCA) [14] and factor analysis [15], is often computationally fast, but requires elaborate normalization procedures that may systematically distort the count data from sequencing technologies [16, 17]. For example, this has led to confusion about whether zero-inflated distributions are needed to analyze scRNA-seq data. Several recent studies have argued this is unnecessary; the high number of zeros is consistent with a simpler Poisson or negative binomial count distribution in both scRNA-seq [18, 19, 20] and ST [21]. To avoid normalization and its pitfalls, DR approaches such as scVI [22], CPLVM [23], and GLM-PCA [24] operate directly on raw counts of unique molecular identifiers (UMIs) by assuming appropriate likelihoods such as the Poisson or negative binomial.

1.2 Gaussian process models

Historically, GPs have been widely used in environmental applications with spatial structure [25]. In the genomics (ST) context, spatialDE [26] fits univariate GP models to ST data to identify which genes are spatially variable. Other examples of univariate GPs applied to ST data include the Bayesian hierarchical model Splotch [27] and the scalable GPcounts [28]. While these methods make positive steps toward including spatial information in routine ST analyses, they do not provide dimension reduction. Genes do not act in isolation but interact with each other, and expression levels of many genes depend on the cellular neighborhood. This means there is substantial gene-gene correlation due to cellular proximity in multivariate ST data ignored by univariate approaches.

A multivariate approach to spatially-aware dimension reduction for ST data is provided by MEFISTO [29]. The key concept of MEFISTO is to represent the high-dimensional gene expression features as a linear combination of a small number of independent GPs over the spatial domain. This is known in the statistics literature as a linear model of coregionalization (LMC) [30]. Historically, LMC factorization required a conjugate (Gaussian) likelihood for computational tractability, which is not appropriate for ST count data. Following [31], we refer to this model as Gaussian process factor analysis (GPFA). In neuroscience, attempts were made to relax the Gaussian assumption for application to functional MRI data, leading to count-GPFA [32].

Even with conjugate likelihoods and univariate outcomes, exact inference for GPs scales cubically with the number of observations (or spatial locations), which is often prohibitive for ST. For example, the recent Slide-seqV2 protocol can generate tens of thousands of observations [8]. Breakthroughs in variational inference for GPs have greatly improved scalability and enabled non-

conjugate likelihoods through approximate inference with inducing points (IPs; see [33] and [34] for overviews). GPFlow is a popular implementation supporting a variety of likelihoods [35]. MEFISTO also uses the variational IP strategy, and in principle is compatible with nonconjugate likelihoods, but in practice the authors recommend using a Gaussian likelihood [29]. An alternative to IPs using polynomial approximate likelihoods [36] has been proposed for count-GPFA [37].

The latent, low-dimensional spatial factors discovered by LMC variants such as GPFA, count-GPFA, and MEFISTO are real-valued. Thus, they may be thought of as spatial analogs of PCA (when the data likelihood is Gaussian) and GLM-PCA (for non-Gaussian data likelihoods). In all cases, latent factors are combined linearly to predict the outcomes. We refer to the weights in these linear combinations as *loadings*, and note that, in the models described above, these loadings are assumed to be real-valued as well. We will use the terms real-valued spatial factorization (RSF) and factor analysis (FA) to refer to these spatial and nonspatial models, respectively. Both RSF and FA models tend to produce dense loadings, but MEFISTO counteracts this with sparsity-promoting priors on the loadings matrix. Sparse loadings are more interpretable than dense because, through nonzero values in the loadings, they assign a small number of relevant features to each component, rather than matching every feature to every component with nonzero weights.

1.3 Nonnegativity

Another way to generate sparse loadings is to constrain the entire model to be nonnegative. In the non-spatial context, nonnegative matrix factorization (NMF) [38] and latent Dirichlet allocation (LDA) [39] are widely used to produce interpretable low-dimensional factorizations of high-dimensional count data [40] including scRNA-seq [41, 42] and ST [43, 44]. To quantify uncertainty, a Bayesian prior can be placed on latent factors and a Poisson or negative binomial data likelihood included to lead to probabilistic NMF (PNMF). The advantage of nonnegative models like PNMF over real-valued alternatives is that, for geometric reasons, they produce parts-based representations rather than holistic representations. Each learned factor from a real-valued model tends to be a linear combination of all the true factors. On the other hand, a nonnegative model would separate out the factors distinctly into what we refer to as a "parts-based" representation; the corresponding loadings are generally sparse as a result of this parts-based representation including only a small number of features in the definition of each of the factors.

Incorporating nonnegativity constraints into spatial models is not straightforward, since GPs are inherently real-valued. Important prior work by [45] proposed GPP-NMF, which is NMF with GP priors. Our approach differs from GPP-NMF in that we use variational inference rather than maximum a posteriori point estimation. Our model can flexibly handle large numbers of irregularly spaced or missing spatial observations, and we automatically learn all hyperparameters during model fitting rather than manually tuning them.

The contributions of this work are threefold. First, we develop nonnegative spatial factorization (NSF), a model that allows spatially-aware dimension reduction using a Gaussian process prior over the spatial locations and with a Poisson or negative binomial likelihood for count data. Second, we combine this spatially-aware dimension reduction with nonspatial factors in a NSF hybrid model (NSFH) to partition variability into the spatial and nonspatial sources. Finally, we identify appropriate GP kernels and develop inference methods for the kernel parameters and latent variables to enable computationally tractable fitting of large field-of-view ST data.

2 Supplemental Notes

2.1 Nonnegative spatial factorization inference

2.1.1 Evidence lower bound objective (ELBO) function

The posterior distribution of NSF cannot be computed in closed form, so we resort to approximate inference using a variational distribution. We assume a set of inducing point locations z_m indexed by $m = 1, \dots, M$. If $M = N$ we set z_m to be the spatial coordinates X. Otherwise, for $M < N$ we set z_m to be the center points of a k-mean clustering (with $k = M$) applied to X. Let $u_{ml} = f_l(z_m)$ be the inducing points, i.e., the Gaussian process evaluation of the inducing locations. We are

interested in inference of the posterior of the latent variables u_{ml} and f_{il}. Temporarily assume loadings w_{jl}, likelihood shape and dispersion parameters, GP prior mean parameters β_l, and kernel hyperparameters θ_l are known. The posterior is given by

$$
\begin{aligned}
p(U,F|\ Y;\ X,Z) &= \frac{p(Y|\ U,F)p(U,F;\ X,Z)}{\int_{U,F} p(Y|\ U,F)p(U,F;\ X,Z)} \\
&= \frac{p(Y|\ F)p(F|\ U;\ X,Z)p(U;\ Z)}{\int_{U,F} p(Y|\ F)p(F|\ U;\ X,Z)p(U;\ Z)}.
\end{aligned}
$$

Note that the likelihood term depends on U only through F, and we have decomposed the joint prior on U,F into a marginal prior of U and a conditional prior of $F|\ U$. Following [46] and [34], the GP prior for inducing points is given by

$$
\begin{aligned}
p(U;\ Z) &= \prod_{l=1}^{L} p(\boldsymbol{u}_l;\ Z) \\
p(\boldsymbol{u}_l;\ Z) &= \mathcal{N}\big(\mu_l(Z),\ K_{uul}\big) \\
\big[K_{uul}\big]_{m,m'} &= k_l\big(\boldsymbol{z}_m, \boldsymbol{z}_{m'}\big).
\end{aligned}
$$

Next, we specify the GP prior for the function values at the observed locations by conditioning on the inducing points.

$$
\begin{aligned}
p(F|\ U;\ X,Z) &= \prod_{l=1}^{L} p(\boldsymbol{f}_l|\ \boldsymbol{u}_l;\ X,Z) \\
p(\boldsymbol{f}_l|\ \boldsymbol{u}_l;\ X,Z) &= \mathcal{N}\big(\boldsymbol{\mu}_{f|\ ul},\ K_{f|\ ul}\big) \\
\boldsymbol{\mu}_{f|\ ul} &= \mu_l(X) + K'_{ufl}K^{-1}_{uul}\big(\boldsymbol{u}_l - \mu_l(Z)\big) \\
K_{f|\ ul} &= K_{ffl} - K'_{ufl}K^{-1}_{uul}K_{ufl} \\
\big[K_{ufl}\big]_{m,i} &= k_l(\boldsymbol{z}_m, \boldsymbol{x}_i) \\
\big[K_{ffl}\big]_{i,i'} &= k_l(\boldsymbol{x}_i, \boldsymbol{x}_{i'}).
\end{aligned}
$$

Note that $K_{uul} \in \mathbb{R}^{M\times M}$, $K_{ffl} \in \mathbb{R}^{N\times N}$, and $K_{ufl} \in \mathbb{R}^{M\times N}$.

We use the following approximation to the true posterior to facilitate variational inference:

$$
\begin{aligned}
q(U,F;\ X,Z) &= p(F|\ U;\ X,Z)q(U;\ Z) \\
q(U;\ Z) &= \prod_{l=1}^{L} q(\boldsymbol{u}_l;\ Z) \\
q(\boldsymbol{u}_l;\ Z) &= \mathcal{N}\big(\boldsymbol{\delta}_l,\ \Omega_l\big).
\end{aligned}
$$

We will later need to draw samples of F from this distribution. This is made easier by analytically marginalizing out U.

$$
\begin{aligned}
q(\boldsymbol{f}_l|\ \boldsymbol{\delta}_l, \Omega_l; X, Z) &= \int_{\boldsymbol{u}_l} p(\boldsymbol{f}_l|\ \boldsymbol{u}_l;\ X,Z)q(\boldsymbol{u}_l;\ Z) \\
&= \mathcal{N}\big(\tilde{\mu}_l,\ \tilde{\Sigma}_l\big),
\end{aligned}
$$

where the marginal mean vector $\tilde{\mu}_l \in \mathbb{R}^N$ and covariance matrix $\tilde{\Sigma}_l \in \mathbb{R}^{N\times N}$ are given by

$$
\begin{aligned}
\tilde{\mu}_l &= \mu_l(X) + K'_{ufl}K^{-1}_{uul}\big(\boldsymbol{\delta}_l - \mu_l(Z)\big) \\
\tilde{\Sigma}_l &= K_{ffl} - K'_{ufl}K^{-1}_{uul}\big(K_{uul} - \Omega_l\big)K^{-1}_{uul}K_{ufl}.
\end{aligned}
$$

Minimizing the KL divergence from the true posterior distribution to the approximating dis-

tribution is equivalent to maximizing the following evidence lower bound (ELBO) [47, 46, 34]:

$$\begin{aligned}
\mathcal{L} &= \mathrm{E}_{q(U,F)}\left[\log\frac{p(Y|\ F)p(F|\ U;\ X,Z)p(U;\ Z)}{q(U,F)}\right] \\
&= \mathrm{E}_{q(U,F)}\left[\log p(Y|\ F)\right] + \mathrm{E}_{q(U,F)}\left[\log\frac{p(F|\ U;\ X,Z)p(U;\ Z)}{p(F|\ U;\ X,Z)q(U;\ Z)}\right] \\
&= (\mathcal{L}_1) - \sum_{l=1}^{L}\mathrm{E}_{q(\boldsymbol{u}_l)}\left[\log\frac{q(\boldsymbol{u}_l;\ Z)}{p(\boldsymbol{u}_l;\ Z)}\right] \\
&= \mathcal{L}_1 - \sum_{l=1}^{L}\mathrm{KL}\left(q(\boldsymbol{u}_l)\ ||\ p(\boldsymbol{u}_l)\right)
\end{aligned}$$

where $\mathcal{L}_1$ is the expected log-likelihood (or reconstruction error). The KL divergence term from prior to approximate posterior has a closed-form expression since both are Gaussian (recall M is the total number of inducing points):

$$\mathrm{KL}\left(q(\boldsymbol{u}_l)\ ||\ p(\boldsymbol{u}_l)\right) = \frac{1}{2}\left[\log\frac{|K_{uul}|}{|\Omega_l|} - M + \mathrm{tr}\left\{K_{uul}^{-1}\Omega_l\right\} + \left(\boldsymbol{\delta}_l - \mu_l(Z)\right)' K_{uul}^{-1}\left(\boldsymbol{\delta}_l - \mu_l(Z)\right)\right].$$

Let $\zeta(y|\ \nu\lambda)$ be the log likelihood of an exponential family such as the Poisson or negative binomial distribution with mean $\nu\lambda$. In particular, for the Poisson distribution, $\zeta(y|\ \nu\lambda) = y\log(\nu\lambda) - \nu\lambda - \log y!$. Let $F[i,:] = (f_{i1},\dots,f_{iL})$. The expected log likelihood term in the ELBO is given by:

$$\begin{aligned}
\mathcal{L}_1 &= \sum_{i=1}^{N}\sum_{j=1}^{J}\mathrm{E}_{q(U,F)}\left[\zeta(y_{ij}|\ \nu_i\lambda_{ij})\right] \\
&= \sum_{i=1}^{N}\sum_{j=1}^{J}\mathrm{E}_{q(F)}\left[\zeta\left(y_{ij}\ \middle|\ \sum_{l=1}^{L} w_{jl}e^{f_{il}}\right)\right] \\
&= \sum_{i=1}^{N}\sum_{j=1}^{J}\mathrm{E}_{q(F[i,:])}\left[\zeta\left(y_{ij}\ \middle|\ \sum_{l=1}^{L} w_{jl}e^{f_{il}}\right)\right].
\end{aligned}$$

The expectation in the above equation is intractable due to the nonlinear log likelihood function $\zeta(\cdot)$. However, we can simplify it in two ways. First, it only depends on U through F, so the marginalized distribution $q(F)$ may be used instead of $q(U,F)$. Second, the log likelihood only depends on the marginal f_{il} terms, as opposed to the multivariate $\boldsymbol{f}_l = (f_{1l},\dots,f_{Nl})$ or the multivariate $F[i,:]$. The approximate posterior distribution is therefore $q(F[i,:]) = \prod_{l=1}^{L} q(f_{il}) = \prod_{l=1}^{L}\mathcal{N}\left([\tilde{\mu}_l]_i,\ \left[\tilde{\Sigma}_l\right]_{i,i}\right)$, where

$$\begin{aligned}
\alpha_l(\boldsymbol{x}_i) &= K_{uul}^{-1}\left[K_{ufl}\right]_{:,i} \\
[\tilde{\mu}_l]_i &= \mu_l(\boldsymbol{x}_i) + \alpha_l(\boldsymbol{x}_i)'\left(\boldsymbol{\delta}_l - \mu_l(Z)\right) \\
\left[\tilde{\Sigma}_l\right]_{i,i} &= k_l(\boldsymbol{x}_i,\boldsymbol{x}_i) - \alpha_l(\boldsymbol{x}_i)'\left(K_{uul} - \Omega_l\right)\alpha_l(\boldsymbol{x}_i).
\end{aligned}$$

Despite these simplifications, the expectation still lacks a closed-form solution and is evaluated by approximation using Monte Carlo (MC) sampling [46]. The MC procedure draws S samples $f_{il}^{(s)} \sim \mathcal{N}\left([\tilde{\mu}_l]_i,\ \left[\tilde{\Sigma}_l\right]_{i,i}\right)$ then evaluates

$$\mathrm{E}_{q(F[i,:])}\left[\zeta\left(y_{ij}\ \middle|\ \sum_{l=1}^{L} w_{jl}e^{f_{il}}\right)\right] \approx \frac{1}{S}\sum_{s=1}^{S}\left[\zeta\left(y_{ij}\ \middle|\ \sum_{l=1}^{L} w_{jl}e^{f_{il}^{(s)}}\right)\right].$$

In practice, we found $S = 3$ to provide a reasonable balance between speed and numerical stability.

2.1.2 Parameter estimation

Using the ELBO as an objective function, we optimize all parameters using the Adam algorithm [48] with gradients computed by automatic differentiation in Tensorflow [49]. This includes the loadings weights w_{jl}, mean function intercepts β_{0l} and slopes $\boldsymbol{\beta}_{1l}$, kernel length scale and amplitude parameters, variational location $\boldsymbol{\delta}_l$ and covariance Ω_l parameters, and any shape or dispersion parameters associated with the likelihood (e.g., for negative binomial and Gaussian distributions). The Adam learning rate was initialized at 0.01 for all models. If a numerical error occurred, all parameters were reset to cached values from 50 iterations previous to the error, and the learning rate was decreased by a factor of 0.5.

To satisfy the nonnegativity constraint on w_{jl}, we use a projected gradient approach. After each optimization step, any values that are negative are truncated to zero. All other parameter constraints are accommodated by monotone transformations. For example, the variational covariance matrices Ω_l must all be positive definite, so we store and use the lower triangular Cholesky decomposition factors instead of the full covariance matrices themselves.

2.1.3 Real-valued spatial factorization inference

The inference procedure for RSF is identical to NSF except we do not exponentiate the sampled $f_{il}^{(s)}$ terms prior to combining with the loadings w_{jl}. Because the loadings are no longer constrained to be greater than or equal to zero, the truncation step is omitted during optimization. To facilitate comparisons with MEFISTO, we focused on a Gaussian likelihood and only applied RSF to normalized data with features centered to have zero mean.

2.2 Nonspatial count factorization inference

To fit PNMF and FA models, we adopt a mean field variational approximation [50] to the posterior distribution of the latent factors:

$$q(f_{il}) = \mathcal{N}(\delta_{il},\ \omega_{il}).$$

Focusing on PNMF, the ELBO is of the form

$$\mathcal{L} = \sum_{i=1}^{N}\sum_{j=1}^{J} \mathrm{E}_{q(F[i,:])}\left[\zeta\left(y_{ij} \;\middle|\; \sum_{l=1}^{L} w_{jl}e^{f_{il}}\right)\right] - \sum_{i=1}^{N}\sum_{l=1}^{L} \mathrm{KL}\left(q(f_{il}) \mid\mid p(f_{il})\right).$$

The expectation in the first term is approximated by MC sampling just as in NSF. The second term involves two univariate Gaussians and has the closed form

$$\mathrm{KL}\left(q(f_{il}) \mid\mid p(f_{il})\right) = \frac{1}{2}\left[\log\frac{s_l^2}{\omega_{il}} - 1 + \frac{\omega_{il}}{s_l^2} + \frac{(\delta_{il} - m_l)^2}{s_l^2}\right].$$

FA has an identical setup to NSF except without exponentiating the sampled $f_{il}^{(s)} \sim q(f_{il})$. Optimization of parameters is the same as in NSF, including the truncation of w_{jl} terms in PNMF.

2.3 Nonnegative spatial factorization hybrid (NSFH) model

Recall $\nu_i\lambda_{ij}$ is the mean of the outcome y_{ij}, which we assume is distributed as some exponential family likelihood, such as Gaussian, negative binomial, or Poisson. The NSFH model is specified as the combination of T spatial factors with $L - T$ nonspatial factors

$$\lambda_{ij} = \sum_{l=1}^{T} w_{jl}e^{f_{il}} + \sum_{l=T+1}^{L} v_{jl}e^{h_{il}}.$$

To estimate the f_{il} terms, we use the same GP prior and variational inducing point approximate posterior as in NSF. To estimate the h_{il} terms, we use the same univariate Gaussian prior and

mean field variational approximate posterior as in PNMF. The ELBO objective function is similar to NSF and PNMF:

$$\mathcal{L} = \sum_{i=1}^{N}\sum_{j=1}^{J} \mathrm{E}_{q(F[i,:],H[i,:])}\left[\zeta\left(y_{ij}\,\middle|\,\sum_{l=1}^{L} w_{jl}e^{f_{il}} + \sum_{l=T+1}^{L} v_{jl}e^{h_{il}}\right)\right]\dots$$

$$\dots - \sum_{l=1}^{L} \mathrm{KL}\left(q(\boldsymbol{u}_l)\mid\mid p(\boldsymbol{u}_l)\right) - \sum_{i=1}^{N}\sum_{l=1}^{L} \mathrm{KL}\left(q(h_{il})\mid\mid p(h_{il})\right).$$

Due to the mean-field formulation, the variational distributions factorize over components:

$$q(F[i,:], H[i,:]) = \prod_{l=1}^{T} q(f_{il}) \prod_{l=T+1}^{L} q(h_{il}).$$

Thus, we approximated the expectation by independent MC samples of $f_{il}^{(s)} \sim q(f_{il})$ and $h_{il}^{(s)} \sim q(h_{il})$. The remaining two KL divergence terms are identical to those in NSF and PNMF and have the same closed form. We optimize all parameters using the same techniques described for NSF and PNMF.

3 Supplemental Discussion

A possible future application of NSFH is denoising. If signal is expected to be spatially correlated while noise is spatially uncorrelated, NSFH could be applied to separate the two. However, this assumption did not seem warranted for the data considered here.

A substantial limitation of the models studied here is the reliance on Euclidean distance as the metric for the GP kernel over the spatial domain. While this was appropriate for the particular datasets we explored, other ST datasets more closely resemble manifolds. An example is the embryonic tissue profiled by [51]. Under such conditions, standard GP kernels are inappropriate [52]. The recently proposed manifold GP [53] and graph GP [54] seem promising as alternatives. Either of these could be substituted for the standard GPs in our RSF, NSF, and NSFH models.

One difficulty with latent factor models is choosing the appropriate number of components. Even in the case of PCA, the choice of dimensionality is somewhat subjective, and heuristics are often used, for example the resampling based "jackstraw" procedure [55]. In our experiments, we varied the number of components for each model and quantified the extent to which this affected metrics like predictive accuracy. A more rigorous approach could be to place a prior on the number of components. However, this would increase the computational complexity of model fitting. Another alternative could be to use the evidence lower bound as a proxy for the marginal likelihood and perform an approximation to Bayesian model comparison [22, 23].

Historically, two major challenges for working with ST data have included integration with single-cell RNA-seq references [56, 57] and deconvolving observations that incorporate multiple cells [58, 59]. Addressing these will be an important future direction for research into nonnegative spatial factor models. Indeed, in our experiments, we observed markedly different results on the Visium dataset, which did not have single-cell resolution, compared to the Slide-seqV2 and XYZeq datasets. However, we anticipate that ongoing improvements in ST protocols will increase the number of genes detected per location while improving the spatial resolution to single-cell or even subcellular levels [5, 6] and maintaining a wide field-of-view.

All of the spatial models we considered were based on linear combinations of GPs with variational inference using inducing points [33, 34]. While this technique has greatly improved GP scalability by enabling minibatching and nonconjugate likelihoods, the computational complexity still scales cubically with the number of inducing points. Promising future directions for GP inference include the harmonic kernel decomposition [60], nearest-neighbor GPs [25, 61], and random Fourier features [62, 63]. While linearity and nonnegativity are advantageous for interpretability [64], multivariate spatial factor models can also be formulated using nonlinear deep GPs [65]. Spatially aware dimension reduction without GPs has been proposed using hidden Markov random fields (HMRF) coupled with NMF as in the SPICEMIX method [66].

We have focused on the application of nonnegative spatial factor models to genomics data. However, both NSF and NSFH are relevant to other types of multivariate spatial or temporal data. Examples include forestry and environmental remote sensing [67], wearable devices [68], and neuroscience [69, 70].

4 Supplemental Figures

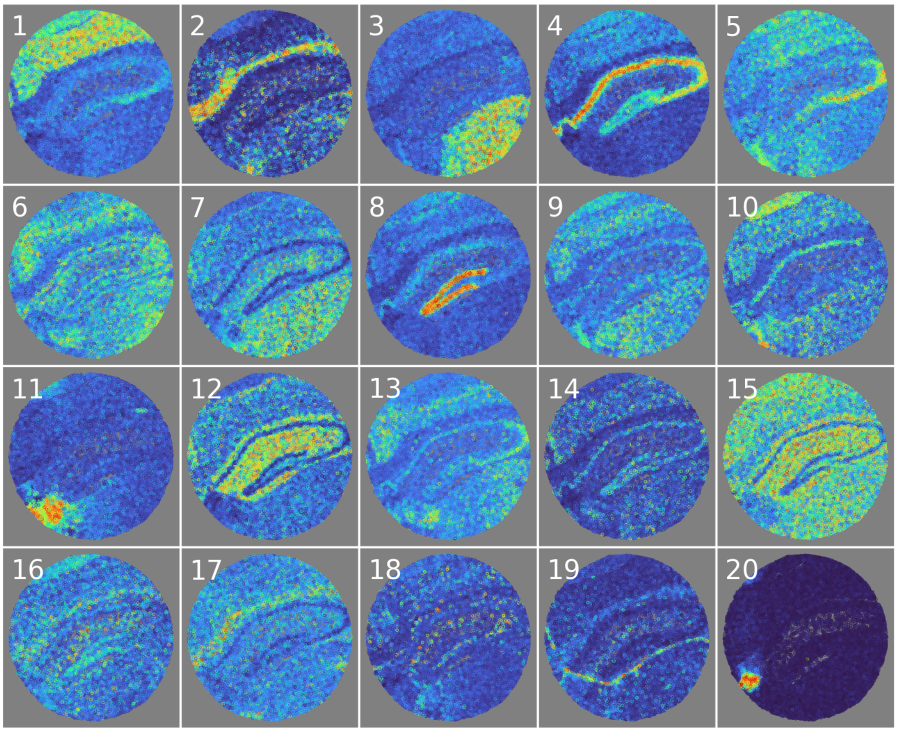

(a) probabilistic nonnegative matrix factorization (PNMF)

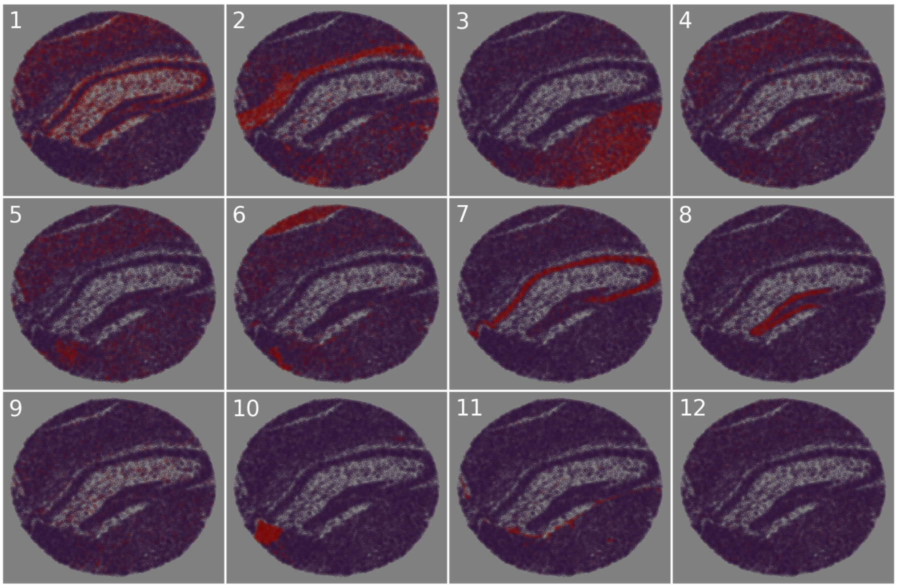

(b) Scanpy clustering

Figure S1: Nonspatial factor models applied to Slide-seqV2 mouse hippocampus gene expression data. Field-of-view is a coronal section with left indicating the medial direction and right the lateral direction. (a) nonnegative factor model, (b) unsupervised clustering using Scanpy

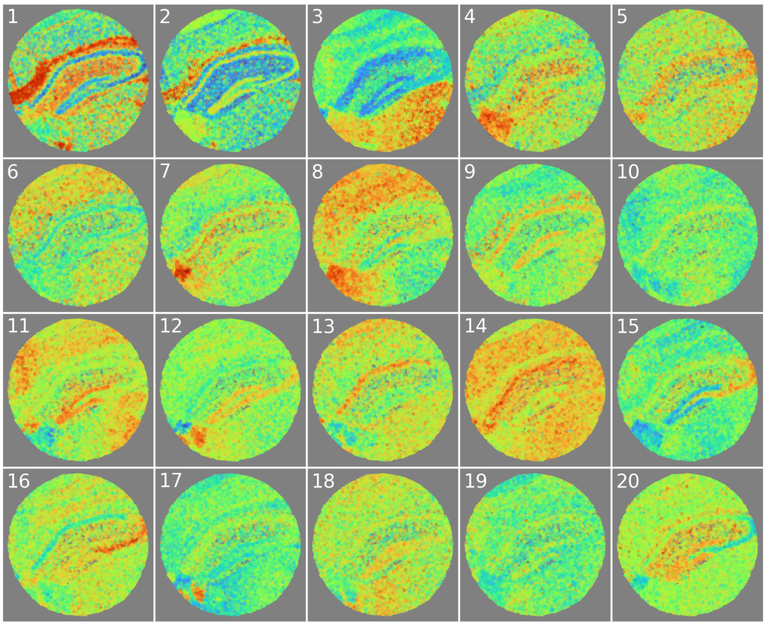

(a) factor analysis (FA)

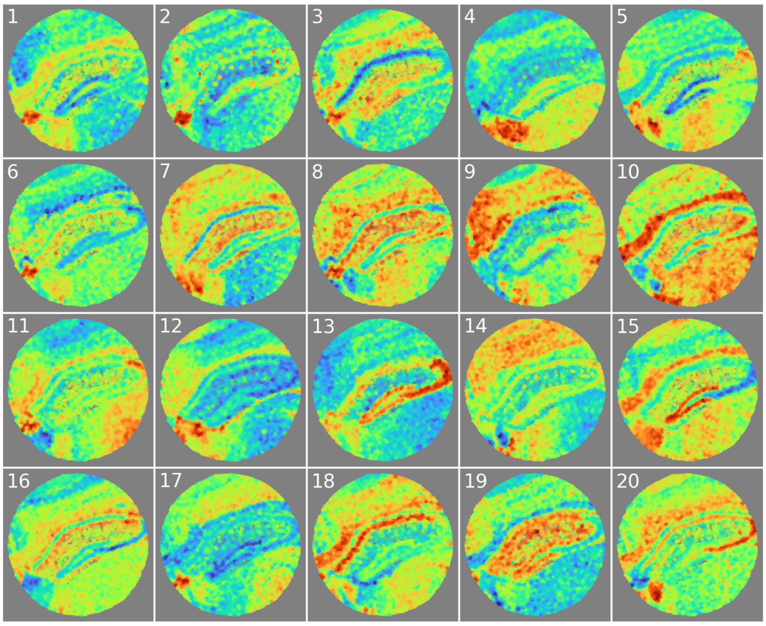

(b) real-valued spatial factorization (RSF)

Figure S2: Real-valued factor models applied to Slide-seqV2 mouse hippocampus gene expression data. Field-of-view is a coronal section with left indicating the medial direction and right the lateral direction. (a) nonspatial factor model, (b) spatial factor model.

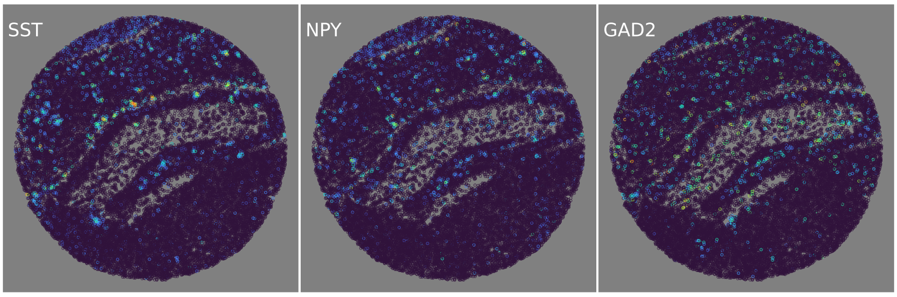

(a) Slide-seqV2 mouse hippocampus

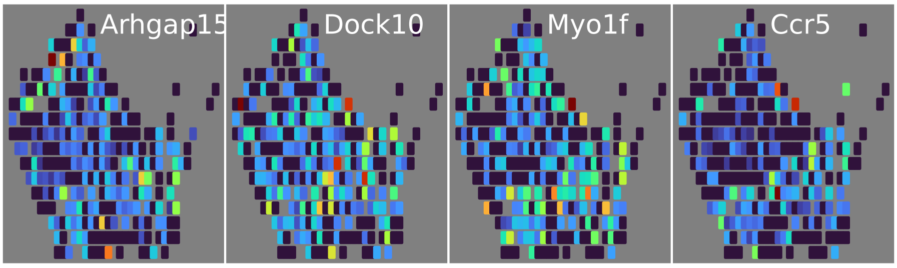

(b) XYZeq mouse liver/tumor

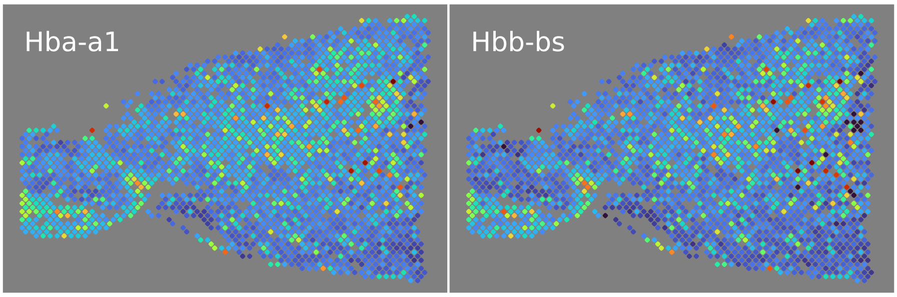

(c) Visium mouse brain

Figure S3: Examples of genes identified as spatially variable by Hotspot, but assigned low spatial importance scores by nonnegative spatial factorization hybrid model.

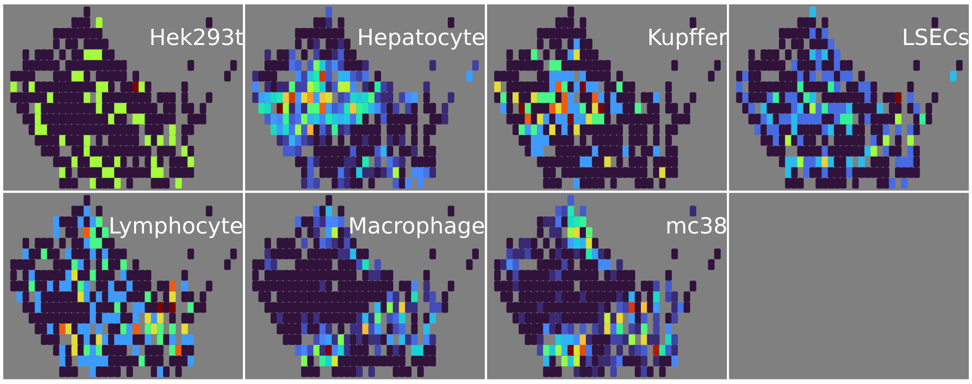

Figure S4: Cell type counts per spatial location in the XYZeq mouse/liver dataset. Annotations from the original authors.

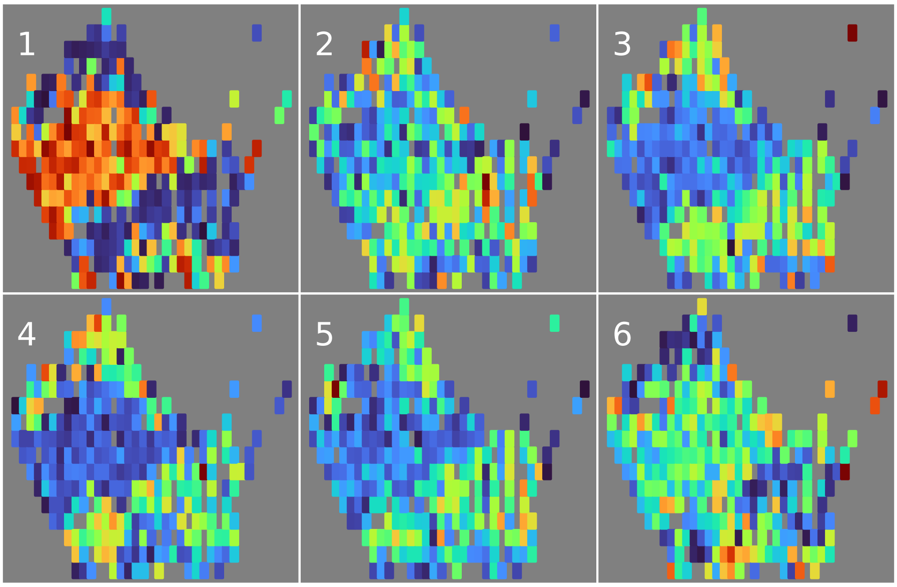

(a) probabilistic nonnegative matrix factorization (PNMF)

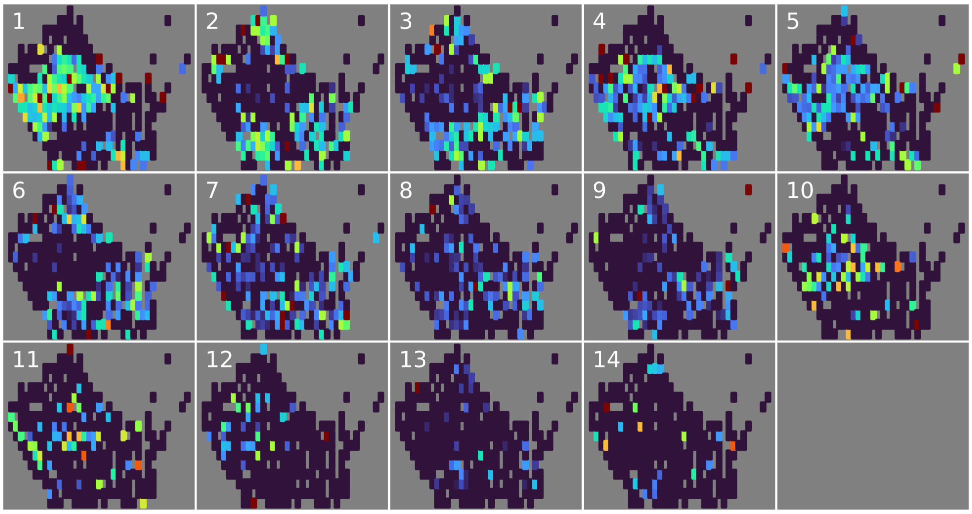

(b) Scanpy clustering

Figure S5: Nonspatial factor models applied to XYZeq mouse liver/tumor gene expression data. (a) nonnegative factor model, (b) unsupervised clustering using Scanpy. Values of multiple cells with same spatial location were averaged together.

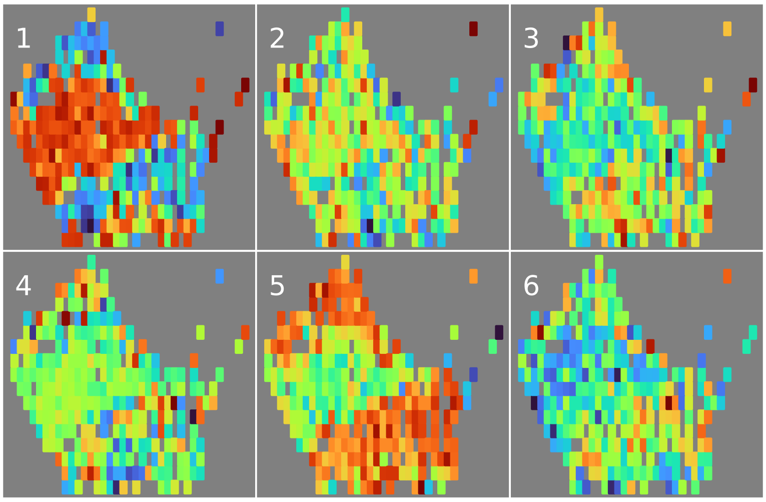

(a) factor analysis (FA)

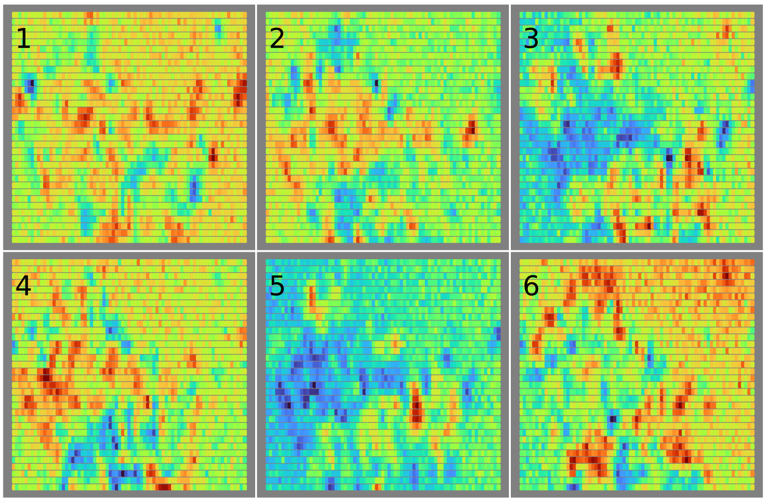

(b) real-valued spatial factorization (RSF)

Figure S6: Real-valued factor models applied to XYZeq mouse liver/tumor gene expression data. (a) nonspatial factor model, values of multiple cells with same spatial location averaged together, (b) spatial factor model with unique values per location.

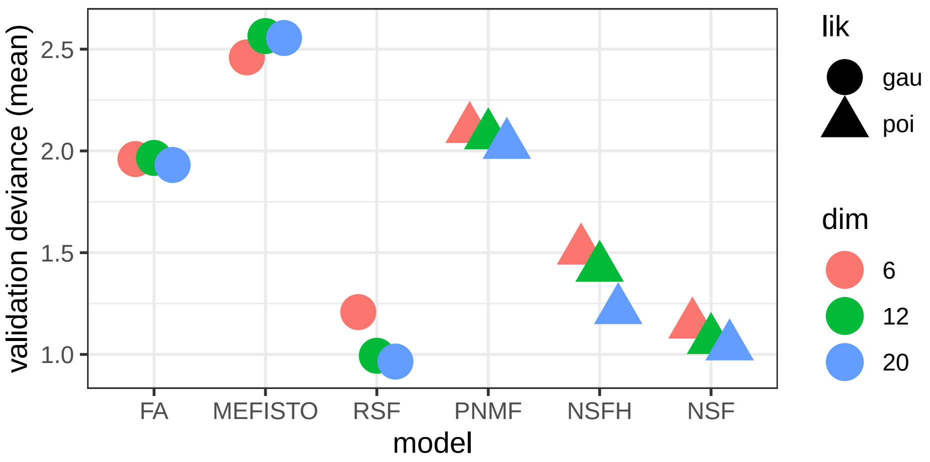

Figure S7: Benchmarking spatial and nonspatial factor models on Visium mouse brain gene expression data. Lower deviance indicates better generalization accuracy. 20% of observations were held out for validation as opposed to 5% elsewhere.

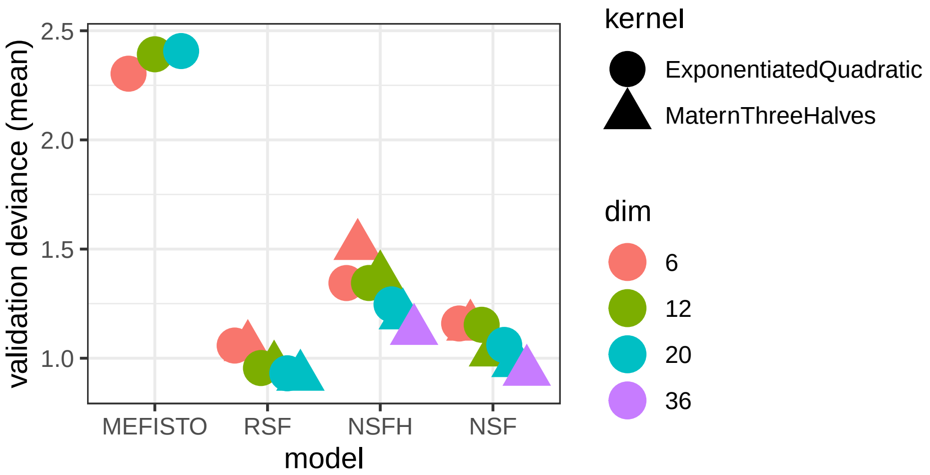

Figure S8: Replacing Matérn(3/2) kernel with exponentiated quadratic (EQ) does not affect generalization accuracy of spatial factor models on Visium mouse brain gene expression data. Lower predictive deviance indicates better generalization accuracy. All models used 1,000 inducing points. dim: number of latent dimensions (components), RSF: real-valued spatial factorization, NSF: nonnegative spatial factorization, NSFH: NSF hybrid model.

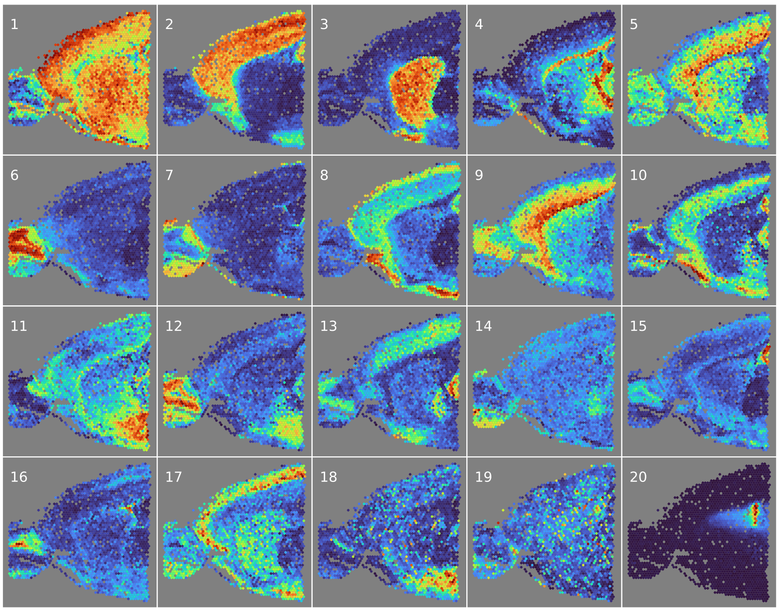

(a) probabilistic nonnegative matrix factorization (PNMF)

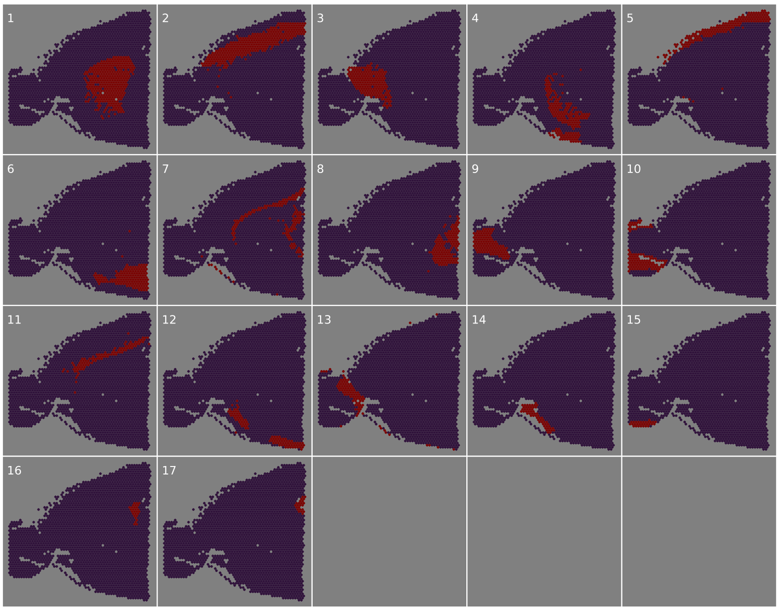

(b) Scanpy clustering

Figure S9: Nonspatial factor models applied to Visium mouse brain gene expression data. Field-of-view is an anterior sagittal section. (a) nonnegative factor model, (b) unsupervised clustering using Scanpy

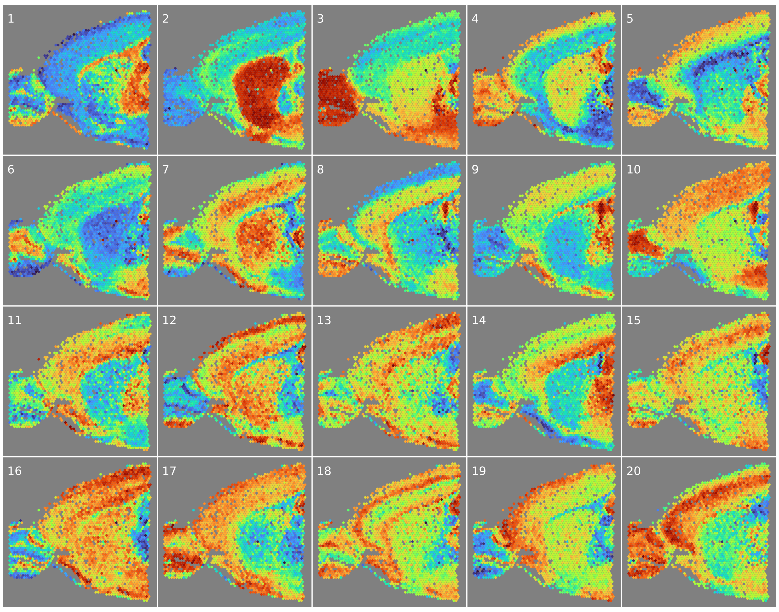

(a) factor analysis (FA)

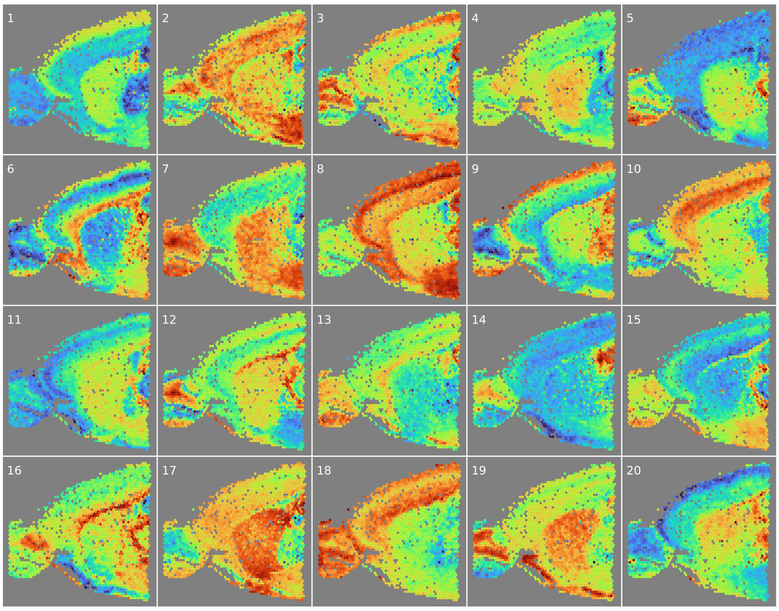

(b) real-valued spatial factorization (RSF)

Figure S10: Real-valued factor models applied to Visium mouse brain gene expression data. Field-of-view is an anterior sagittal section. (a) nonspatial factor model, (b) spatial factor model.

5 Supplemental Tables

Table S1: Summary of probabilistic factor models for high-dimensional spatial count data. An X in the nonnegative, spatial, or nonspatial column indicates whether the model includes that type of latent factors. Likelihoods are listed with the default choice of each model first. gau: Gaussian or normal distribution, poi: Poisson, nb: negative binomial.

**abbrev**	**model**	**nonnegative**	**spatial**	**nonspatial**	**likelihoods**
FA	factor analysis			X	gau
PNMF	probabilistic nonnegative matrix factorization	X		X	poi, nb, gau
MEFISTO	MEFISTO		X		gau, poi
RSF	real-valued spatial factorization		X		gau
NSF	nonnegative spatial factorization	X	X		poi, nb, gau
NSFH	nonnegative spatial factorization hybrid	X	X	X	poi, nb, gau

Table S2: Spatial transcriptomics datasets. Slide-seqV2 and XYZeq provide single-cell resolution, whereas each Visium observation is an average of multiple cells. XYZeq combines multiple observations at each spatial location. obs: number of observations, resolution: center-to-center distance between spatial locations, FOV: field of view area.

**first author**	**year**	**tissue**	**protocol**	**obs**	**locations**	**resolution**	**FOV**
Stickels	2021	hippocampus	Slide-seqV2	36,536	36,536	10 µm	$7.4\,mm^2$
Lee	2021	liver/ tumor	XYZeq	2,700	289	500 µm	$87.6\,mm^2$
10x Genomics	2020	brain- anterior	Visium	2,487	2,487	100 µm	$42.3\,mm^2$

Table S3: Nonnegative spatial factorization hybrid model (NSFH) identifies biologically distinct components in Slide-seqV2 mouse hippocampus.

dim	type	brain regions	cell types	genes	GO biological processes
1	spat	choroid plexus of third ventricle	Choroid plexus cells	*TTR, ENPP2, IFI27, TRPM3, STK39*	T cell migration, cellular response to chemokine
2	spat	thalamus	Interneurons	*PRKCD, TNNT1, RAMP3, NTNG1, PDP1*	regulation of presynaptic cytosolic calcium ion concentration, proteoglycan metabolic process
3	spat	CA1-3 (Ammon's Horn) pyramidal layer	Neurons	*HPCA, NEUROD6, CRYM, WIPF3, CPNE6*	positive regulation of dendritic spine morphogenesis, postsynaptic modulation of chemical synaptic transmission
4	spat	cerebral cortex	Neurons	LAMP5, *3110035E14RIK, STX1A, MEF2C, EGR1*	myeloid leukocyte differentiation, hormone biosynthetic process
5	spat	fiber tracts/ corpus callosum	Oligodendrocytes	*CLDN11, MAL, MAG, PLP1, MOG*	myelination, central nervous system myelination
6	spat	medial habenula (thalamus)	Neurons	*NWD2, TAC2, CALB2, NECAB2, ZCCHC12*	steroid biosynthetic process, sex differentiation
7	spat	CA strata and dentate gyrus molecular layer	Astrocytes	*DDN, SLC1A3, CST3, PSD, CABP7*	learning, response to amino acid
8	spat	dentate gyrus granule layer	Neurons	*LRRTM4, STXBP6, SLC8A2, 2010300C02RIK, PLXNA4*	central nervous system projection neuron axonogenesis, regulation of cytoskeleton organization
9	spat	multiple	Astrocytes	*SLC6A11, SPARC, SLC4A4, KCNJ10, ATP1A2*	negative regulation of blood coagulation, cellular amino acid catabolic process
10	spat	meninges	Meningeal cells	*PTGDS, GFAP, APOD, FABP7, FXYD1*	nitric oxide mediated signal transduction, epithelial cell proliferation
1	nsp		Neurons	*MEG3, SNHG11, MIAT, CPNE7, TTC14*	mRNA processing, RNA splicing
2	nsp		GABAergic neurons	*SST, NPY, GAD2, GAD1, CNR1*	neurotransmitter metabolic process, negative regulation of catecholamine secretion
3	nsp		Neurons	*CHGA, RAB3C, HSPA4L, CKMT1, SYT4*	chemical synaptic transmission, regulation of short-term neuronal synaptic plasticity
4	nsp		GABAergic neurons	*PVALB, VAMP1, CPLX1, MT-ND1, SCRT1*	mitochondrial respiratory chain complex I assembly, aerobic respiration
5	nsp		Astrocytes	*MT-RNR2, MT-RNR1, 2900052N01RIK, MAP2, MT-ND5*	electron transport coupled proton transport, response to nicotine
6	nsp		Astrocytes	*GM3764, MALAT1, GPC5, LSAMP, TRPM3*	cell adhesion, synaptic membrane adhesion
7	nsp		Neurons	*NEFM, NEFH, MAP1B, VAMP1, SLC24A2*	cell adhesion, intermediate filament cytoskeleton organization
8	nsp		Interneurons	*NPTXR, SYN2, STMN2, NCALD, YWHAH*	mitotic cell cycle, thyroid gland development
9	nsp		Neurons	*NRG3, FGF14, CSMD1, DLG2, KCNIP4*	social behavior, positive regulation of synapse assembly
10	nsp			*MIR6236, LARS2, CMSS1, HEXB, CAMK1D*	translation, positive regulation of signal transduction by p53 class mediator

Table S4: Hotspot clustering of genes in Slide-seqV2 mouse hippocampus dataset.

cluster	genes	GO biological processes
1	*MBP, KIF5A, FTH1, MOBP, PLP1*	reproduction, regulation of DNA recombination
2	*PTGDS, MT-ND1, MT-ND4, MT-CYTB, CST3*	reproduction, regulation of DNA recombination
3	*HPCA, NRGN, TMSB4X, PPP3CA, OLFM1*	reproduction, regulation of DNA recombination
4	*PCP4, TNNT1, PRKCD, PCP4L1, ADARB1*	proteoglycan metabolic process, sensory perception of chemical stimulus
5	*CAMK2N1, MEF2C, TSHZ2, 3110035E14RIK, LAMP5*	reproduction, regulation of DNA recombination
6	*SNAP25, VSNL1, CALM1, LMO4, ATP1B1*	reproduction, regulation of DNA recombination
7	*CALB2, TAC2, NECAB2, ZIC1, NWD2*	reproduction, regulation of DNA recombination
8	*CPLX1, NEFH, NEFM, RGS4, VAMP1*	intermediate filament cytoskeleton organization, postsynaptic cytoskeleton organization
9	*DDN, CAMK2A, PSD, CPLX2, CABP7*	dendrite development, filopodium assembly
10	*CALB1, 6330403A02RIK, FAM19A5, RNF112, SPON1*	actin filament-based movement, plasma membrane repair
11	*RPL13A, RPL4, TUBB2B, RPS24, GAS5*	translation, cytoplasmic translation
12	*TTR, ENPP2, IFI27, TRPM3, BSG*	embryo implantation, fatty acid catabolic process
13	*UQCRH, COX6C, ATP5K, NDUFA13, ATP5G3*	mitochondrial respiratory chain complex I assembly, mitochondrial electron transport, ubiquinol to cytochrome c
14	*ATPIF1, ELAVL2, CHD3OS, FKBP3, NME1*	cellular amino acid metabolic process, regulation of autophagy of mitochondrion
15	*CHST2, LSAMP, NRXN1, FAM213B, IDS*	regulation of presynapse assembly, synaptic membrane adhesion
16	*MARCKS, H3F3B, YBX1, HMGN1, RSRP1*	negative regulation of mRNA splicing, via spliceosome, mRNA splicing, via spliceosome
17	*HOMER2, GIT1, COX8A, PALM, SNPH*	dendritic spine development, regulation of cytokinesis
18	*NFIX, INF2, NCAM1, ANK3, ARHGEF2*	cell morphogenesis, positive regulation of DNA replication
19	*GRM8, STMN1, TUBB5, PINK1, KIF1A*	histone deacetylation, positive regulation of receptor internalization

Table S5: Nonnegative spatial factorization hybrid model (NSFH) identifies biologically distinct components in XYZeq mouse liver.

dim	type	cell types	genes	GO biological processes
1	spat	Hepatocytes	*HNF1AOS1, CPS1, CYP2E1, AKR1C6, MUG2*	cellular amino acid catabolic process, xenobiotic metabolic process
2	spat		*IL31RA, SEMA5A, TRPM3, PLCD1, FOXP4*	positive regulation of cholesterol esterification, inclusion body assembly
3	spat	Macrophages	*LGALS1, S100A6, S100A4, RPL30, KLF6*	translation, cytoplasmic translation
1	nsp	Fibroblasts	*KIF26B, MEDAG, LAMA4, NGF, COL1A2*	regulation of cellular response to vascular endothelial growth factor stimulus, collagen fibril organization
2	nsp		*HMGA2, SLC35F1, FAM19A1, TENM4, HS3ST5*	cell fate specification, specification of animal organ identity
3	nsp	Macrophages	*ARHGAP15, DOCK10, MYO1F, LY86, HCK*	negative regulation of leukocyte apoptotic process, negative regulation of immune response

Table S6: Hotspot clustering of genes in XYZeq mouse liver/tumor dataset.

**cluster**	**genes**	**GO biological processes**
1	*PIGR, SEMA5A, ALB, TRF, PPARA*	reproduction, ribosomal small subunit assembly
2	*SNX24, CMSS1, FOXP4, CD5L, RNF152*	reproduction, ribosomal small subunit assembly
3	*MSN, RPL32, ANP32B, RPSA, AHNAK*	cytoplasmic translation, translation
4	*DPYSL3, SPARC, ZFPM2, FBN1, RPLP1*	reproduction, ribosomal small subunit assembly
5	*ACTB, CTSS, CCR5, DOCK10, TGFBI*	antigen processing and presentation of exogenous peptide antigen, negative regulation of leukocyte apoptotic process
6	*MITF, RPS21, ITM2B, LPCAT2, BTAF1*	histone H4 acetylation, non-membrane-bounded organelle assembly

Table S7: Convergence failures in fitting models to the Visium mouse brain dataset. The ExponentiatedQuadratic kernel led to numerical instabilities with large numbers of inducing points (IPs).

**kernel**	**IPs**	**model**	**likelihood**	**total runs**	**converged**	**fraction**
MaternThreeHalves	500	NSF	poi	8	8	1
MaternThreeHalves	500	NSFH	poi	8	8	1
MaternThreeHalves	500	RSF	gau	3	3	1
MaternThreeHalves	1000	NSF	poi	8	8	1
MaternThreeHalves	1000	NSFH	poi	8	8	1
MaternThreeHalves	1000	RSF	gau	3	3	1
MaternThreeHalves	2363	NSF	poi	8	8	1
MaternThreeHalves	2363	NSFH	poi	9	9	1
MaternThreeHalves	2363	RSF	gau	3	3	1
ExponentiatedQuadratic	500	NSF	poi	6	6	1
ExponentiatedQuadratic	500	NSFH	poi	6	6	1
ExponentiatedQuadratic	500	RSF	gau	3	3	1
ExponentiatedQuadratic	1000	NSF	poi	6	6	1
ExponentiatedQuadratic	1000	NSFH	poi	6	6	1
ExponentiatedQuadratic	1000	RSF	gau	3	3	1
ExponentiatedQuadratic	2363	NSF	poi	6	0	0
ExponentiatedQuadratic	2363	NSFH	poi	6	0	0
ExponentiatedQuadratic	2363	RSF	gau	3	0	0

Table S8: Nonnegative spatial factorization hybrid model (NSFH) identifies biologically distinct components in Visium mouse brain.

**dim**	**type**	**brain regions**	**cell types**	**genes**	**GO biological processes**
1	spat	multiple		*IGKC, COX6A2, TNNC1, CABP7, S100A9*	mitochondrial electron transport, NADH to ubiquinone, mitochondrial respiratory chain complex I assembly
2	spat	cerebral cortex	Interneurons	*CCK, DKK3, STX1A, NRN1, RTN4R*	axonogenesis, positive regulation of behavioral fear response
3	spat	basal ganglia	Neurons	*GPR88, PDE10A, RGS9, PPP1R1B, PENK*	response to amphetamine, striatum development
4	spat	fiber tracts/ corpus callosum	Oligodendrocytes	*PLP1, MAL, MOBP, MAG, CLDN11*	myelination, central nervous system myelination
5	spat	olfactory granule layer	Neurons	*GNG4, GPSM1, CPNE4, SHISA8, MYO16*	embryonic limb morphogenesis, proximal/distal pattern formation
6	spat	multiple	Interneurons	*NPTXR, CCN3, SLC30A3, RASL10A, LMO3*	intracellular signal transduction, regulation of catalytic activity
7	spat	outer olfactory bulb	Interneurons	*S100A5, DOC2G, CDHR1, CALB2, FABP7*	mesoderm formation, cellular response to glucose stimulus
8	spat	inner cerebral cortex	Neurons	*HS3ST2, IGHM, CCN2, IGSF21, NR4A2*	isoprenoid biosynthetic process, cholesterol biosynthetic process
9	spat	multiple	GABAergic neurons	*GAD1, SLC32A1, HAP1, STXBP6, CPNE7*	neurotransmitter metabolic process, regulation of gamma-aminobutyric acid secretion
10	spat	choroid plexus of lateral ventricle	Choroid plexus cells	*TTR, ECRG4, ENPP2, KL, 2900040C04RIK*	hormone transport, retinol metabolic process
1	nsp		GABAergic neurons	*PVALB, KCNAB3, CPLX1, VAMP1, SYT2*	positive regulation of potassium ion transmembrane transporter activity, neuromuscular process
2	nsp	hypothalamus	Neurons	*BAIAP3, NNAT, HPCAL1, LYPD1, RESP18*	neurotrophin TRK receptor signaling pathway, muscle fiber development
3	nsp		Neurons	*NEFM, LGI2, DNER, PLCXD2, CARTPT*	regulation of synaptic vesicle fusion to presynaptic active zone membrane, neuronal action potential propagation
4	nsp	hippocampus	Neurons	*WIPF3, CPNE6, RGS14, ARPC5, CABP7*	Arp2/3 complex-mediated actin nucleation, calcineurin-NFAT signaling cascade
5	nsp		Neurons	*HS3ST4, RAB26, CLSTN2, TLE4, SNCA*	vacuolar acidification, protein glycosylation
6	nsp		Vascular fibroblasts	*LARS2, GM42418, VTN, PTN, NPY*	establishment of epithelial cell polarity, plasma lipoprotein particle organization
7	nsp		Neurons	*PLXND1, VSTM2L, CALB1, RGS7, MGAT5B*	heterophilic cell-cell adhesion via plasma membrane cell adhesion molecules, short-term memory
8	nsp		GABAergic neurons	*SST, NPY, RESP18, NOS1, PDYN*	neuropeptide signaling pathway, regulation of the force of heart contraction
9	nsp		Astrocytes	*TUBB2B, GM3764, NTRK2, MFGE8, PLPP3*	complement activation, negative regulation of growth
10	nsp		Erythrocytes	*HBA-A1, HBA-A2, HBB-BT, HBB-BS, ALAS2*	oxygen transport, cellular oxidant detoxification

Table S9: Hotspot clustering of genes in Visium mouse brain dataset.

cluster	genes	GO biological processes
1	*PLP1, MBP, FTH1, MOBP, TRF*	reproduction, ribosomal large subunit assembly
2	*PPP1R1B, RASD2, GPR88, SCN4B, PDE10A*	reproduction, ribosomal large subunit assembly
3	*GAD1, GNG4, CSDC2, PCBP3, CKB*	reproduction, ribosomal large subunit assembly
4	*CCK, SNAP25, VSNL1, 1110008P14RIK, BASP1*	axonogenesis, synaptic vesicle fusion to presynaptic active zone membrane
5	*EEF1A1, RPS12, RPS29, RPLP1, RPS9*	reproduction, ribosomal large subunit assembly
6	*FABP7, S100A5, APOD, APOE, PTN*	plasma lipoprotein particle organization, establishment of epithelial cell polarity
7	*MEG3, SNHG11, 6330403K07RIK, HAP1, SCG2*	neurotrophin TRK receptor signaling pathway, cellular response to BMP stimulus
8	*ADCY1, VXN, COX8A, EFHD2, IGHM*	response to lithium ion, spontaneous neurotransmitter secretion
9	*NRGN, CAMK2A, CAMK2N1, CALM1, MEF2C*	phosphatidylinositol phosphate biosynthetic process, negative regulation of heart contraction
10	*GM42418, LARS2, AGT, SPARC, DBI*	reproduction, ribosomal large subunit assembly
11	*IGFBP2, ENPP2, COL9A3, KL, ECRG4*	oxygen transport, erythrocyte development
12	*TUBB2A, TUBA1A, CPNE6, CACNB3, GPRIN1*	reproduction, ribosomal large subunit assembly
13	*VAMP2, SYN1, DLGAP1, STXBP1, PPIA*	regulation of cardiac muscle contraction by regulation of the release of sequestered calcium ion, regulation of cell communication
14	*CALM2, ITPR1, PRKCB, SELENOW, CTXN1*	reproduction, ribosomal large subunit assembly
15	*GABRA1, IGFBP4, ALDOA, CCN3, CHGB*	reproduction, ribosomal large subunit assembly
16	*NSG2, CAMK2B, NDRG4, NCDN, C1QTNF4*	mRNA splice site selection, face development
17	*ATP1B1, CPLX1, ATP1A3, NEFL, NEFM*	proton transmembrane transport, electron transport chain
18	*GNAS, TUBA1B, GAPDH, HSPA8, MAP1B*	vacuolar acidification, lysosome organization
19	*R3HDM1, ADGRB2, CACNA2D1, ZDHHC8, BRINP1*	synaptic vesicle maturation, vocalization behavior
20	*NNAT, GRIA1, CPNE7, FXYD6, SYN2*	nucleoside monophosphate metabolic process, nucleobase metabolic process
21	*APP, KIF5C, SPOCK1, CKMT1, BEND6*	positive regulation of neuron differentiation, negative regulation of endopeptidase activity
22	*RASGRF2, CAMK2N2, SLC1A2, RIMS3, CUX2*	positive regulation of glucose import, canonical glycolysis
23	*SLC32A1, GAD2, ABAT, KLHL13, NRSN2*	neurotransmitter metabolic process, negative regulation of wound healing
24	*RPL3, MAPK3, CDO1, RPL10A, NACA*	positive regulation of translation, positive regulation of histone acetylation
25	*RAPGEF4, SEPT7, TUBB4B, NUDT4, KCNA6*	negative regulation of peptidyl-tyrosine phosphorylation, peptidyl-tyrosine dephosphorylation
26	*CPLX3, CCN2, IGSF21, NXPH3, NR4A2*	cellular polysaccharide biosynthetic process, positive regulation of glial cell proliferation
27	*PVALB, SIX3, RAMP3, VAMP1, UNC13C*	positive regulation of fat cell differentiation, female gonad development
28	*CABP7, CRYM, TMSB4X, HS3ST4, WIPF3*	cholesterol biosynthetic process, Arp2/3 complex-mediated actin nucleation

References

[1] Moses L, Pachter L. Museum of Spatial Transcriptomics. Nature Methods. 2022 May;19(5):534–546.

[2] Editors. Method of the Year 2020: Spatially Resolved Transcriptomics. Nature Methods. 2021 Jan;18(1):1–1.

[3] Maynard KR, Collado-Torres L, Weber LM, Uytingco C, Barry BK, Williams SR, et al. Transcriptome-Scale Spatial Gene Expression in the Human Dorsolateral Prefrontal Cortex. Nature Neuroscience. 2021 Feb;p. 1–12.

[4] Verma A, Jena SG, Isakov DR, Aoki K, Toettcher JE, Engelhardt BE. A Self-Exciting Point Process to Study Multicellular Spatial Signaling Patterns. Proceedings of the National Academy of Sciences. 2021 Aug;118(32).

[5] Eng CHL, Lawson M, Zhu Q, Dries R, Koulena N, Takei Y, et al. Transcriptome-Scale Super-Resolved Imaging in Tissues by RNA seqFISH+. Nature. 2019 Apr;568(7751):235–239.

[6] Xia C, Fan J, Emanuel G, Hao J, Zhuang X. Spatial Transcriptome Profiling by MERFISH Reveals Subcellular RNA Compartmentalization and Cell Cycle-Dependent Gene Expression. Proceedings of the National Academy of Sciences. 2019 Sep;116(39):19490–19499.

[7] Vickovic S, Eraslan G, Salmén F, Klughammer J, Stenbeck L, Schapiro D, et al. High-Definition Spatial Transcriptomics for in Situ Tissue Profiling. Nature Methods. 2019 Sep;p. 1–4.

[8] Stickels RR, Murray E, Kumar P, Li J, Marshall JL, Di Bella DJ, et al. Highly Sensitive Spatial Transcriptomics at Near-Cellular Resolution with Slide-seqV2. Nature Biotechnology. 2021 Mar;39(3):313–319.

[9] Wolf FA, Angerer P, Theis FJ. SCANPY: Large-Scale Single-Cell Gene Expression Data Analysis. Genome Biology. 2018 Feb;19(1):15.

[10] Butler A, Hoffman P, Smibert P, Papalexi E, Satija R. Integrating Single-Cell Transcriptomic Data across Different Conditions, Technologies, and Species. Nature Biotechnology. 2018 Apr;.

[11] Sun S, Zhu J, Ma Y, Zhou X. Accuracy, Robustness and Scalability of Dimensionality Reduction Methods for Single-Cell RNA-seq Analysis. Genome Biology. 2019 Dec;20(1):269.

[12] Palla G, Spitzer H, Klein M, Fischer D, Schaar AC, Kuemmerle LB, et al. Squidpy: A Scalable Framework for Spatial Omics Analysis. Nature Methods. 2022 Jan;p. 1–8.

[13] Dries R, Zhu Q, Dong R, Eng CHL, Li H, Liu K, et al. Giotto: A Toolbox for Integrative Analysis and Visualization of Spatial Expression Data. Genome Biology. 2021 Mar;22(1):78.

[14] Hotelling H. Analysis of a Complex of Statistical Variables into Principal Components. Journal of Educational Psychology. 1933;24(6):417–441.

[15] Bartholomew DJ, Knott M, Moustaki I. Latent Variable Models and Factor Analysis: A Unified Approach. John Wiley & Sons; 2011.

[16] Hicks SC, Townes FW, Teng M, Irizarry RA. Missing Data and Technical Variability in Single-Cell RNA-sequencing Experiments. Biostatistics. 2018 Oct;19(4):562–578.

[17] Townes FW, Hicks SC, Aryee MJ, Irizarry RA. Feature Selection and Dimension Reduction for Single-Cell RNA-Seq Based on a Multinomial Model. Genome Biology. 2019 Dec;20(1):295.

[18] Svensson V. Droplet scRNA-seq Is Not Zero-Inflated. Nature Biotechnology. 2020 Jan;p. 1–4.

[19] Kim TH, Zhou X, Chen M. Demystifying “Drop-Outs” in Single-Cell UMI Data. Genome Biology. 2020 Aug;21(1):196.

[20] Sarkar A, Stephens M. Separating Measurement and Expression Models Clarifies Confusion in Single-Cell RNA Sequencing Analysis. Nature Genetics. 2021 Jun;53(6):770–777.

[21] Zhao P, Zhu J, Ma Y, Zhou X. Modeling Zero Inflation Is Not Necessary for Spatial Transcriptomics. Genome Biology. 2022 May;23(1):118.

[22] Lopez R, Regier J, Cole MB, Jordan MI, Yosef N. Deep Generative Modeling for Single-Cell Transcriptomics. Nature Methods. 2018 Dec;15(12):1053–1058.

[23] Jones A, Townes FW, Li D, Engelhardt BE. Contrastive Latent Variable Modeling with Application to Case-Control Sequencing Experiments. The Annals of Applied Statistics. 2022 Sep;16(3):1268–1291.

[24] Townes FW, Street K, Yeung J. Glmpca: Dimension Reduction of Non-Normally Distributed Data; 2019.

[25] Finley AO, Datta A, Cook BD, Morton DC, Andersen HE, Banerjee S. Efficient Algorithms for Bayesian Nearest Neighbor Gaussian Processes. Journal of Computational and Graphical Statistics. 2019 Apr;28(2):401–414.

[26] Svensson V, Teichmann SA, Stegle O. SpatialDE: Identification of Spatially Variable Genes. Nature Methods. 2018 May;15(5):343–346.

[27] Äijö T, Maniatis S, Vickovic S, Kang K, Cuevas M, Braine C, et al. Splotch: Robust Estimation of Aligned Spatial Temporal Gene Expression Data. bioRxiv. 2019 Sep;p. 757096.

[28] BinTayyash N, Georgaka S, John ST, Ahmed S, Boukouvalas A, Hensman J, et al. Non-Parametric Modelling of Temporal and Spatial Counts Data from RNA-seq Experiments. Bioinformatics. 2021 Nov;37(21):3788–3795.

[29] Velten B, Braunger JM, Argelaguet R, Arnol D, Wirbel J, Bredikhin D, et al. Identifying Temporal and Spatial Patterns of Variation from Multimodal Data Using MEFISTO. Nature Methods. 2022 Jan;p. 1–8.

[30] Gelfand AE, Schmidt AM, Banerjee S, Sirmans CF. Nonstationary Multivariate Process Modeling through Spatially Varying Coregionalization. Test. 2004 Dec;13(2):263–312.

[31] Yu BM, Cunningham JP, Santhanam G, Ryu SI, Shenoy KV, Sahani M. Gaussian-Process Factor Analysis for Low-Dimensional Single-Trial Analysis of Neural Population Activity. In: Koller D, Schuurmans D, Bengio Y, Bottou L, editors. Advances in Neural Information Processing Systems 21. Curran Associates, Inc.; 2009. p. 1881–1888.

[32] Zhao Y, Park IM. Variational Latent Gaussian Process for Recovering Single-Trial Dynamics from Population Spike Trains. Neural Computation. 2017 Mar;29(5):1293–1316.

[33] Leibfried F, Dutordoir V, John ST, Durrande N. A Tutorial on Sparse Gaussian Processes and Variational Inference. arXiv:201213962 [cs, stat]. 2021 Feb;.

[34] van der Wilk M, Dutordoir V, John ST, Artemev A, Adam V, Hensman J. A Framework for Interdomain and Multioutput Gaussian Processes. arXiv:200301115 [cs, stat]. 2020 Mar;.

[35] Matthews AGdG, van der Wilk M, Nickson T, Fujii K, Boukouvalas A, Leon-Villagra P, et al. GPflow: A Gaussian Process Library Using TensorFlow. Journal of Machine Learning Research. 2017;18(40):1–6.

[36] Huggins J, Adams RP, Broderick T. PASS-GLM: Polynomial Approximate Sufficient Statistics for Scalable Bayesian GLM Inference. In: Guyon I, Luxburg UV, Bengio S, Wallach H, Fergus R, Vishwanathan S, et al., editors. Advances in Neural Information Processing Systems 30. Curran Associates, Inc.; 2017. p. 3611–3621.

[37] Keeley S, Zoltowski D, Yu Y, Smith S, Pillow J. Efficient Non-conjugate Gaussian Process Factor Models for Spike Count Data Using Polynomial Approximations. In: International Conference on Machine Learning. PMLR; 2020. p. 5177–5186.

[38] Lee DD, Seung HS. Learning the Parts of Objects by Non-Negative Matrix Factorization. Nature. 1999 Oct;401(6755):788–791.

[39] Blei DM, Ng AY, Jordan MI. Latent Dirichlet Allocation. J Mach Learn Res. 2003 Mar;3:993–1022.

[40] Carbonetto P, Sarkar A, Wang Z, Stephens M. Non-Negative Matrix Factorization Algorithms Greatly Improve Topic Model Fits. arXiv:210513440 [cs, stat]. 2021 May;.

[41] Elyanow R, Dumitrascu B, Engelhardt BE, Raphael BJ. netNMF-sc: Leveraging Gene-Gene Interactions for Imputation and Dimensionality Reduction in Single-Cell Expression Analysis. Genome Research. 2020 Jan;p. gr.251603.119.

[42] Sherman TD, Gao T, Fertig EJ. CoGAPS 3: Bayesian Non-Negative Matrix Factorization for Single-Cell Analysis with Asynchronous Updates and Sparse Data Structures. BMC Bioinformatics. 2020 Oct;21(1):453.

[43] Berglund E, Maaskola J, Schultz N, Friedrich S, Marklund M, Bergenstråhle J, et al. Spatial Maps of Prostate Cancer Transcriptomes Reveal an Unexplored Landscape of Heterogeneity. Nature Communications. 2018 Jun;9(1):2419.

[44] Zeira R, Land M, Strzalkowski A, Raphael BJ. Alignment and Integration of Spatial Transcriptomics Data. Nature Methods. 2022 May;p. 1–9.

[45] Schmidt MN, Laurberg H. Nonnegative Matrix Factorization with Gaussian Process Priors. Computational Intelligence and Neuroscience. 2008 Apr;2008:e361705.

[46] Salimbeni H, Deisenroth M. Doubly Stochastic Variational Inference for Deep Gaussian Processes. In: Advances in Neural Information Processing Systems; 2017. p. 4588–4599.

[47] Hensman J, Fusi N, Lawrence ND. Gaussian Processes for Big Data. arXiv:13096835 [cs, stat]. 2013 Sep;.

[48] Kingma D, Ba J. Adam: A Method for Stochastic Optimization. arXiv:14126980 [cs]. 2014 Dec;.

[49] Abadi M, Barham P, Chen J, Chen Z, Davis A, Dean J, et al. TensorFlow: A System for Large-Scale Machine Learning. In: Proceedings of the 12th USENIX Symposium on Operating Systems Design and Implementation; 2016. p. 265–283.

[50] Blei DM, Kucukelbir A, McAuliffe JD. Variational Inference: A Review for Statisticians. Journal of the American Statistical Association. 2017 Apr;112(518):859–877.

[51] Lohoff T, Ghazanfar S, Missarova A, Koulena N, Pierson N, Griffiths JA, et al. Integration of Spatial and Single-Cell Transcriptomic Data Elucidates Mouse Organogenesis. Nature Biotechnology. 2022 Jan;40(1):74–85.

[52] Dunson DB, Wu HT, Wu N. Diffusion Based Gaussian Processes on Restricted Domains. arXiv:201007242 [math, stat]. 2020 Oct;.

[53] Borovitskiy V, Terenin A, Mostowsky P, Deisenroth (he/him) M. Matérn Gaussian Processes on Riemannian Manifolds. In: Advances in Neural Information Processing Systems. vol. 33. Curran Associates, Inc.; 2020. p. 12426–12437.

[54] Borovitskiy V, Azangulov I, Terenin A, Mostowsky P, Deisenroth M, Durrande N. Matérn Gaussian Processes on Graphs. In: International Conference on Artificial Intelligence and Statistics. PMLR; 2021. p. 2593–2601.

[55] Macosko EZ, Basu A, Satija R, Nemesh J, Shekhar K, Goldman M, et al. Highly Parallel Genome-wide Expression Profiling of Individual Cells Using Nanoliter Droplets. Cell. 2015 May;161(5):1202–1214.

[56] Lopez R, Nazaret A, Langevin M, Samaran J, Regier J, Jordan MI, et al. A Joint Model of Unpaired Data from scRNA-seq and Spatial Transcriptomics for Imputing Missing Gene Expression Measurements. arXiv:190502269 [cs, q-bio, stat]. 2019 May;.

[57] Verma A, Engelhardt B. A Bayesian Nonparametric Semi-Supervised Model for Integration of Multiple Single-Cell Experiments. bioRxiv. 2020 Jan;p. 2020.01.14.906313.

[58] Cable DM, Murray E, Zou LS, Goeva A, Macosko EZ, Chen F, et al. Robust Decomposition of Cell Type Mixtures in Spatial Transcriptomics. Nature Biotechnology. 2021 Feb;p. 1–10.

[59] Lopez R, Li B, Keren-Shaul H, Boyeau P, Kedmi M, Pilzer D, et al. DestVI Identifies Continuums of Cell Types in Spatial Transcriptomics Data. Nature Biotechnology. 2022 Sep;40(9):1360–1369.

[60] Sun S, Shi J, Wilson AG, Grosse R. Scalable Variational Gaussian Processes via Harmonic Kernel Decomposition. arXiv:210605992 [cs, stat]. 2021 Jun;.

[61] Wu L, Pleiss G, Cunningham J. Variational Nearest Neighbor Gaussian Processes. arXiv:220201694 [cs, stat]. 2022 Feb;.

[62] Hensman J, Durrande N, Solin A. Variational Fourier Features for Gaussian Processes. The Journal of Machine Learning Research. 2017 Jan;18(1):5537–5588.

[63] Gundersen GW, Zhang MM, Engelhardt BE. Latent Variable Modeling with Random Features. arXiv:200611145 [cs, stat]. 2020 Jun;.

[64] Svensson V, Gayoso A, Yosef N, Pachter L. Interpretable Factor Models of Single-Cell RNA-seq via Variational Autoencoders. Bioinformatics. 2020;.

[65] Wu A, Nastase SA, Baldassano CA, Turk-Browne NB, Norman KA, Engelhardt BE, et al. Brain Kernel: A New Spatial Covariance Function for fMRI Data. NeuroImage. 2021 Dec;245:118580.

[66] Chidester B, Zhou T, Alam S, Ma J. SPICEMIX: Integrative Single-Cell Spatial Modeling of Cell Identity. bioRxiv; 2022.

[67] Taylor-Rodriguez D, Finley AO, Datta A, Babcock C, Andersen HE, Cook BD, et al. Spatial Factor Models for High-Dimensional and Large Spatial Data: An Application in Forest Variable Mapping. Statistica Sinica. 2019;29:1155–1180.

[68] Straczkiewicz M, James P, Onnela JP. A Systematic Review of Smartphone-Based Human Activity Recognition Methods for Health Research. npj Digital Medicine. 2021 Oct;4(1):1–15.

[69] Wu A, Roy NA, Keeley S, Pillow JW. Gaussian Process Based Nonlinear Latent Structure Discovery in Multivariate Spike Train Data. In: Guyon I, Luxburg UV, Bengio S, Wallach H, Fergus R, Vishwanathan S, et al., editors. Advances in Neural Information Processing Systems 30. Curran Associates, Inc.; 2017. p. 3496–3505.

[70] Foti NJ, Fox EB. Statistical Model-Based Approaches for Functional Connectivity Analysis of Neuroimaging Data. Current Opinion in Neurobiology. 2019 Apr;55:48–54.
