# Supplementary material for: Nonnegative spatial factorization applied to spatial genomics
Source: Nat Methods. 2022 Dec 31;20(2):229–38. doi: 10.1038/s41592-022-01687-w (PMC9911348; doi:10.1038/s41592-022-01687-w)
Supplement: Supplementary file 2 — Reporting Summary. [file 41592_2022_1687_MOESM2_ESM.pdf]

nature research

Corresponding author(s): Barbara E. Engelhardt

Last updated by author(s): Sep 30, 2022

Reporting Summary

Nature Research wishes to improve the reproducibility of the work that we publish. This form provides structure for consistency and transparency in reporting. For further information on Nature Research policies, see our Editorial Policies and the Editorial Policy Checklist.

Statistics

For all statistical analyses, confirm that the following items are present in the figure legend, table legend, main text, or Methods section.

n/a	Confirmed	
☐	☒	The exact sample size (*n*) for each experimental group/condition, given as a discrete number and unit of measurement
☐	☒	A statement on whether measurements were taken from distinct samples or whether the same sample was measured repeatedly
☐	☒	The statistical test(s) used AND whether they are one- or two-sided<br>*Only common tests should be described solely by name; describe more complex techniques in the Methods section.*
☒	☐	A description of all covariates tested
☐	☒	A description of any assumptions or corrections, such as tests of normality and adjustment for multiple comparisons
☒	☐	A full description of the statistical parameters including central tendency (e.g. means) or other basic estimates (e.g. regression coefficient) AND variation (e.g. standard deviation) or associated estimates of uncertainty (e.g. confidence intervals)
☒	☐	For null hypothesis testing, the test statistic (e.g. *F*, *t*, *r*) with confidence intervals, effect sizes, degrees of freedom and *P* value noted<br>*Give P values as exact values whenever suitable.*
☐	☒	For Bayesian analysis, information on the choice of priors and Markov chain Monte Carlo settings
☒	☐	For hierarchical and complex designs, identification of the appropriate level for tests and full reporting of outcomes
☐	☒	Estimates of effect sizes (e.g. Cohen's *d*, Pearson's *r*), indicating how they were calculated

Our web collection on statistics for biologists contains articles on many of the points above.

Software and code

Policy information about availability of computer code

Data collection	We used Python 3.8.10 and R 4.1.0 to access publicly available datasets from previous studies.
Data analysis	We implemented all models using Python 3.8.10, tensorflow 2.5.0, and tensorflow probability 0.13.0. Other Python packages used include scanpy 1.8.0, squidpy 1.1.0, scikit-learn 0.24.2, pandas 1.2.5,numpy 1.19.5, and scipy 1.7.0. We used the MEFISTO implementation from the mofapy2 Python package, installed from the GitHub development branch at commit 8f6ffcb5b18d22b3f44ff2a06bcb92f2806afed0. Graphics were generated using either matplotlib 3.4.2 in Python or ggplot2 3.3.5 (Wickham, 2016) in R (version 4.1.0). The R packages Seurat 0.4.3 (Hao et al., 2021), SeuratData 0.2.1, and SeuratDisk 0.0.0.9019 were used for some initial data manipulations. Computationally-intensive model fitting was done on Princeton's Della cluster. Each model was assigned 12 CPU cores. We provided the following total memory per dataset: 180 Gb for Slide-seq V2, 72 Gb for Visium, and 48 Gb for XYZeq. Bioconductor (version 3.13) R packages biomaRt (version 2.48.0) and topGO (version 2.44.0) were used for gene ontology analyses. Code for reproducing the analyses of this manuscript is available from https://github.com/willtownes/nsf-paper and an installable python package is available from https://github.com/willtownes/spatial-factorization-py.

For manuscripts utilizing custom algorithms or software that are central to the research but not yet described in published literature, software must be made available to editors and reviewers. We strongly encourage code deposition in a community repository (e.g. GitHub). See the Nature Research guidelines for submitting code & software for further information.

Data

Policy information about availability of data

All manuscripts must include a data availability statement. This statement should provide the following information, where applicable:

- Accession codes, unique identifiers, or web links for publicly available datasets
- A list of figures that have associated raw data
- A description of any restrictions on data availability

All data used in this manuscript are from public sources. The Visium mouse brain dataset "Mouse Brain Serial Section 1 (Sagittal-Anterior)" was downloaded from https://cf.10xgenomics.com/ samples/spatial-exp/1.1.0/V1_Mouse_Brain_Sagittal_Anterior/ V1_Mouse_Brain_Sagittal_ Anterior_filtered_feature_bc_matrix.h5 and https://cf.10xgenomics.com/samples/spatial-exp/ 1.1.0/ V1_Mouse_Brain_Sagittal_Anterior/V1_Mouse_Brain_Sagittal_Anterior_spatial.tar. gz. The Slide-seqV2 mouse hippocampus dataset was originally produced by [9]. We obtained it through the SeuratData R package (version 0.2.1) [28]. Raw data is available from https:// singlecell.broadinstitute.org/single_cell/study/SCP815/highly-sensitive-spatial-transcriptomics-at study-summary. The XYZeq mouse liver data was obtained from the Gene Expression Omnibus [30], accession number GSE164430. We focused on sample liver slice L20C1, which was featured in the original publication, and downloaded it as an H5AD file. The spatial coordinates were provided by the original authors. Additional databases used in the study included Biomart (http://www.biomart.org/), Panglao (https://panglaodb.se/), and scfind (https: //scfind.sanger.ac.uk/).

Field-specific reporting

Please select the one below that is the best fit for your research. If you are not sure, read the appropriate sections before making your selection.

☒ Life sciences ☐ Behavioural & social sciences ☐ Ecological, evolutionary & environmental sciences

For a reference copy of the document with all sections, see nature.com/documents/nr-reporting-summary-flat.pdf

Life sciences study design

All studies must disclose on these points even when the disclosure is negative.

Sample size	We used only public data, so no sample size calculations were performed ahead of time. Sample sizes reflected inclusion of as many observations as possible from original studies subject to standard quality control filtering. Sample sizes for simulations were set to mimic typical values found in public datasets.
Data exclusions	Visium mouse brain: observations (spots) with total counts less than 100 or mitochondrial counts greater than 20% were excluded. Slide-seqV2 mouse hippocampus: observations (spots) with total counts less than 100 or mitochondrial counts greater than 20% were excluded.
Replication	While we did not generate any data, all code is provided openly to enable others to replicate our results.
Randomization	We did not generate new data for this study, so we did not randomize the data. We did use different random seeds in simulations to create a range of scenarios for assessing the relative performance of different models.
Blinding	Blinding was not necessary for this study, since we did not generate any new data to test a hypothesis.

Reporting for specific materials, systems and methods

We require information from authors about some types of materials, experimental systems and methods used in many studies. Here, indicate whether each material, system or method listed is relevant to your study. If you are not sure if a list item applies to your research, read the appropriate section before selecting a response.

Materials & experimental systems

n/a	Involved in the study	
☒	☐	Antibodies
☒	☐	Eukaryotic cell lines
☒	☐	Palaeontology and archaeology
☒	☐	Animals and other organisms
☒	☐	Human research participants
☒	☐	Clinical data
☒	☐	Dual use research of concern

Methods

n/a	Involved in the study	
☒	☐	ChIP-seq
☒	☐	Flow cytometry
☒	☐	MRI-based neuroimaging
